# Involvement of the anterior cingulate cortex in time-based prospective memory task monitoring: An EEG analysis of brain sources using Independent Component and Measure Projection Analysis

**DOI:** 10.1371/journal.pone.0184037

**Published:** 2017-09-01

**Authors:** Gabriela Cruz, Pablo Burgos, Kerry Kilborn, Jonathan J. Evans

**Affiliations:** 1 School of Psychology, University of Glasgow, Glasgow, Scotland; 2 Institute of Health and Wellbeing, University of Glasgow, Glasgow, Scotland; 3 Escuela de Terapia Ocupacional, Universidad de Chile, Santiago, Chile; 4 Departamento de Kinesiología, Universidad de Chile, Santiago, Chile; University of British Columbia, CANADA

## Abstract

**Objective:**

Time-based prospective memory (PM), remembering to do something at a particular moment in the future, is considered to depend upon self-initiated strategic monitoring, involving a retrieval mode (sustained maintenance of the intention) plus target checking (intermittent time checks). The present experiment was designed to explore what brain regions and brain activity are associated with these components of strategic monitoring in time-based PM tasks.

**Method:**

24 participants were asked to reset a clock every four minutes, while performing a foreground ongoing word categorisation task. EEG activity was recorded and data were decomposed into source-resolved activity using Independent Component Analysis. Common brain regions across participants, associated with retrieval mode and target checking, were found using Measure Projection Analysis.

**Results:**

Participants decreased their performance on the ongoing task when concurrently performed with the time-based PM task, reflecting an active retrieval mode that relied on withdrawal of limited resources from the ongoing task. Brain activity, with its source in or near the anterior cingulate cortex (ACC), showed changes associated with an active retrieval mode including greater negative ERP deflections, decreased theta synchronization, and increased alpha suppression for events locked to the ongoing task while maintaining a time-based intention. Activity in the ACC was also associated with time-checks and found consistently across participants; however, we did not find an association with time perception processing *per se*.

**Conclusion:**

The involvement of the ACC in both aspects of time-based PM monitoring may be related to different functions that have been attributed to it: strategic control of attention during the retrieval mode (distributing attentional resources between the ongoing task and the time-based task) and anticipatory/decision making processing associated with clock-checks.

## Introduction

Many daily life activities involve execution of non-routine delayed intentions, such as attending a meeting, taking a child to the doctor or calling a friend on her birthday. These are referred to as Prospective Memory (PM) tasks. Failures are common in people with neurological conditions and therefore a key issue in cognitive rehabilitation [1, 2] and an increasing focus of research [3–5].

Research in prospective memory has focused on event-based prospective memory tasks (e.g. remembering to give a message to a colleague when you see her in the corridor), in which an environmental event signals the moment to retrieve the intention. Less attention has been paid to time-based prospective memory tasks, when the retrieval of the intention is signalled by the passage of time and the intention is initiated in the absence of an external event (e.g. remember to call someone in 10 minutes). A key issue is the extent to which successful performance of PM tasks relies on attentional monitoring in order to detect the appropriate moment to implement a PM intention [6]. For instance, it has been proposed that time-based prospective memory tasks are more demanding and require higher levels of monitoring compared to event-based prospective memory tasks [7]. But most theories of PM have not explicitly considered time-based PM tasks [8–10] and little is known about monitoring mechanisms in time-based PM tasks.

The term monitoring is commonly used in PM tasks that rely on internal strategies for successful performance [10, 11]. Monitoring is an effortful process, drawing on limited attentional resources, therefore impacting the performance of other ‘ongoing’ tasks being performed whilst maintaining a PM task intention, relative to the performance of the ongoing task in the absence of any PM intentions. This PM task interference effect [12, 13] is often expressed as increased reaction times for ongoing trials in PM experiments, but it can also be expressed as decreased accuracy [14, 15]. From a behavioural perspective, Guynn’s Monitoring Theory was the first to specifically incorporate time-based PM tasks. The theory defines two components of monitoring: (1) the retrieval mode: a sustained process that reflects the active maintenance of the intention; and (2) target checking: an intermittent process associated with the evaluation of environmental cues that may signal the execution of the intention [11, 16]. Separating the two components in a behavioural paradigm is challenging [17], but studies examining the neural correlates of monitoring can offer valuable insights.

Neuroimaging studies of PM have revealed the relevance of distinct sub-regions of Brodmann area 10 for the performance of PM tasks (see [18] for a review), providing evidence for the role of rostral prefrontal cortex in the active maintenance of delayed intentions [19], in event- and time-based PM tasks [20]. For a review including other brain regions involved in PM tasks see [19]. Functional magnetic resonance imaging and positron emission tomography experiments focus on cumulative changes, and can reveal the involvement of particular brain regions during the performance of PM tasks. However, these imaging modalities lack the temporal resolution to study, for example, brain activity trial-locked to PM events alone (excluding the ongoing task events) or neural processes occurring in the range of milliseconds, as may be the case for transient components of monitoring, e.g. target checking. Conversely, electroencephalography (EEG) has greater temporal resolution, revealing the timing of the different mechanisms that may be involved in monitoring.

Most studies using EEG to study PM have examined monitoring using event-related potentials (ERPs), i.e. voltage deflection revealed by averaging EEG activity time-locked to a particular type of stimulus. The underlying assumption is that after averaging only the activity relevant for the processing of the stimulus will remain, whereas “random” or unrelated activity will be cancelled out. Research shows great variability in the topography, timing and duration of ERP modulations associated with monitoring [14, 15, 21–28]. Cona et al. [22] published the only study, as far as we know, that has examined ERPs in a time-based PM task. They observed sustained frontal activity–beginning from as early as 130ms and lasting several hundred milliseconds–in both time- and event-based PM tasks. Cona et al. [22] interpreted this result as a neural correlate of a retrieval mode, because it was independent of the type of PM task and it probably reflected increased activity in frontal regions (e.g. BA10) to maintain the intention. All other studies of ERP modulations have used event-based PM tasks. West et al. [15] also described a sustained frontal and posterior slow wave–lasting several seconds and maintained during intervals when no stimuli were present–as a correlate of retrieval mode. On the other hand, target checking has been associated with transient ERP modulations thought to be mediated by increased allocation of attentional resources, e.g. as a frontal positive or negative occipital modulation around 200ms [24, 25, 28] and a positive modulation between 400-600ms [22, 27]. In general, long lasting slow waves have been associated with a retrieval mode and transient ERP activity has been associated with target checking [24, 26], but there are exceptions to this view. West et al. [29] interpreted a sustained frontal positivity/occipital negativity beginning at around 300ms as a correlate of target checking, because it was only observed in trials preceding a PM cue. In addition, Cona et al. [22] described a transient modulation between 130ms and 180ms as a correlate of preparatory attention, incorporated in the concept of the retrieval mode, because the timing of this modulation was too early to be considered target checking. Taking all together–and despite the discrepancies on what can be considered neural correlates of either transient or sustained mechanisms for monitoring–the evidence shows that a variety of processes underlie monitoring depending on the nature of PM task.

Furthermore, when interpreting ERP modulations, we should take into account the fact that electrodes placed over the scalp measure brain activity coming from several to many cortical sources, having broad and strongly overlapping scalp projections [30]. The imprecise spatial resolution of EEG does not allow conclusions regarding what are the neural generators of the ERP modulations reported so far in the PM literature. To deal with this spatial limitation, in a previous study we applied Independent Component Analysis (ICA) and a source localization algorithm [31] to two different event-based PM tasks, embedded in the same semantic ongoing task: one aimed at detecting perceptual PM cues (upper-case letters) and the other aimed at detecting conceptual PM cues (animal words). We found neural correlates of monitoring mechanisms with their probable brain source generators: during the PM block, ERPs time-locked to the ongoing task events showed an enhanced negativity around 200ms, with common occipital generators for perceptual and conceptual PM tasks, but also a distinct left temporal generator (i.e., located in or near BA 22/BA21) for the conceptual PM task. In accordance with previous reports, we interpreted this early modulation as a sign of preparatory attention [22, 25, 28], driven by different brain sources depending on the task. In addition, we found a late correlate of target checking (around 700ms), only for ongoing trials that were also potential conceptual PM cues, with neural generators in areas associated with semantic processing (i.e. in or near BA22) and reading (i.e. in or near BA40).

In the present experiment we aimed to complement our previous study by using the same semantic ongoing task but this time embedding a time-based PM paradigm. We used ICA analysis to explore ERPs at a brain source level (instead of scalp ERPs) and added an Event Related Spectral Perturbations (ERSP) analysis. ERSPs measure the amplitude of spectral changes in different frequency bands in relation to the presentation of an experimental event, revealing brain dynamics not contained in the ERPs [32]. In general, low frequency bands (i.e. theta) tend to increase in amplitude during information processing [33], whereas alpha desynchronization reflects increased levels of cortical activation [34]. In sum, we used EEG (brain sources, voltage and frequency analysis) to investigate the neural correlates of the retrieval mode and target checking in a time-based PM task. The retrieval mode was operationalized as performance of an ongoing task while holding a time-based intention and target checking was operationalized as clock checks. We asked people to reset a clock every four minutes while performing a non-related ongoing task [31]. We hypothesised that some attentional resources [11, 16, 35, 36] would be devoted to monitoring time–in order to identify the moment to execute the intention–instead of evaluating ongoing trials (in contrast to event-based PM tasks where ongoing trials evaluation is essential to detect the PM event). This would be expressed as reduced amplitude of ERP components time locked to ongoing trials during the PM block. ERSPs have not been previously used to study time-based PM tasks, however, based on the literature in event-related spectral changes we expected to find correlates of increased cortical activation and reduced resources to process ongoing task events during the PM block [33, 34]. Regarding the second component of monitoring in Guynn’s theory, target checking, our approach is exploratory, as it has not been previously evaluated using EEG.

The present work contributes to understanding the neural correlates of monitoring proposed by Guynn [16] applied to a time-based prospective memory task. It also reveals brain areas relevant for time-based PM tasks using high-density EEG and how these areas may be involved in the monitoring process.

## Materials and methods

### Participants

Twenty-four university students participated in the study, (mean age = 21 years, SD = 5, 13 females and 11 males) recruited from Glasgow University, all native English speakers, right handed, with no history of neurological disorders and normal/corrected-to-normal vision. They received monetary compensation for their participation. Ethical approval was obtained from the College of Science and Engineering Ethic Committee (CSE01307), and all participants provided informed consent prior to participation.

### Procedure

The experiment took approximately one hour to complete: around 20 minutes to set up the electrode net and adjust impedances, plus approximately 40 minutes to complete the task. In the first part of the task participants completed the ongoing task only, control block (~10 minutes). The second part of the experiment consisted of the same ongoing task plus the time-based prospective memory task, PM block (~25 minutes). We used this design to avoid any possible effect of monitoring that could remain during the control block if performed after the PM block, as some previous research suggests [29, 37].

### Ongoing task

The ongoing task was a continuous 1-back categorisation task, in which participants had to decide if the previous word on the screen belonged to the same semantic category as the current word on the screen. This produced two types of ongoing trials: related words, when the word belonged to the same category; and unrelated words, when the word did not belong to the same category. A list of categories was used based on the updated version of the Battig and Montague (1969) category norms [38]. Participants performed the ongoing task under two conditions: First, they performed the ongoing task only (control block). Second, they performed the ongoing task plus the time-based PM task (PM block). The former comprised 300 trials and the latter 600 trials. Each trial lasted for 2 seconds with each word displayed for 500ms. Participants had frequent breaks after a randomised number of ongoing task events, so they could not use breaks as an indicator of the passage of time.

### Prospective memory task

**Fig 1:**
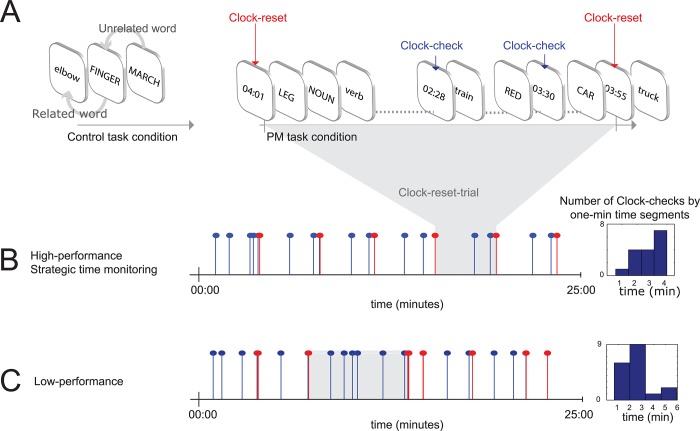
Experimental paradigm and example of performance from two participants. (A) Participants performed the control block, consisting of a continuous 1-back categorisation task (left), followed by the performance of the PM block (ongoing task plus the time-based PM, right). Red arrows indicate clock-resets and blue arrows show clock-checks. A clock-reset-trial is highlighted in grey. (B) Example of a high-performance participant. Blue bars represent clock-checks and red bars represent clock-resets, the frequency of clock-checks increases towards the 4-minute target time (right-hand graph). (C) Example of a low-performance participant. Clock-checks were mainly performed at the beginning of the clock-reset-trial (right-hand graph). Note that the length of clock-reset trials was not fixed but depended on each participant’s performance.

Participants were instructed to reset a clock every four minutes by pressing the first key on a response pad located at their left hand. A digital timer showed up in the centre of the screen for one second every time participants reset the clock, showing the exact time in minutes and seconds counted from the previous clock reset (MM:SS). Participants were also allowed to do clock-checks, which were performed by pressing the second key on the response pad. Clock-checks displayed the same timer on the screen (MM:SS), with the only difference that the clock was not reset (Fig 1). Participants were instructed to be as accurate as they could in the 4-minute clock-reset, while maintaining good performance in the ongoing task. The timer popped up in the centre of the screen masking the ongoing task stimuli, thus every time the clock was displayed they missed a trial of the ongoing task. The reason for masking ongoing trial events with the clock was to encourage participants to check the clock strategically, in order to maintain good performance in the ongoing task. Note that there are no fixed 4-minute clock-reset-trials in the experiment. Each clock-reset-trial started when the clock was reset to zero and lasted until the participant decided to reset the clock to zero again.

### Data acquisition

EEG data were recorded vertex-referenced using a 128-sensor Geodesic Sensor Net (Electrical Geodesics Inc.). The sensor net was soaked in a saline electrolyte solution and adjusted until all pedestals were properly seated on the scalp. Individual sensor impedances were adjusted until they were below 50 kΩ, (in some participants electrodes with impedances below100 kΩ were kept). Data were sampled at 250 Hz with an analog filter bandpass of 0.1–200 Hz. A Macintosh computer running EGI’s Netstation software was used for data collection. E-Prime running on a PC was used for stimulus presentation. Two four-button response pads (one for each hand) were used to collect finger press responses to stimulus events.

### Behavioural analysis

In line with previous studies of time-based prospective memory tasks, efficient monitoring behaviour was defined as a combination of the following three factors [39]: (i) accurate clock resetting, (ii) higher time check frequency towards the end of the target time (four minutes in the present study) and (iii) good performance in the ongoing task while performing the time-based prospective memory task simultaneously.

#### Clock reset accuracy

As participants very rarely reset the clock at exactly the target time, studies in time-based PM typically use an arbitrary limit to define an accurate clock reset, for example giving a range of 20 or 30 seconds around the target time [22, 40, 41]. Here we used non-parametric descriptive statistics to explore performance at single subject level, the advantage of this approach being that we did not use arbitrary limits to define what it is an accurate clock reset. We used boxplots to graphically depict the time variation for clock-reset and clock-checks and defined accuracy based on all participants’ behaviour. When plotting the single subject performance for clock reset accuracy we identified a high variability in the responses, thus we decided to use the median of the clock-reset time across all participants to define two different performance groups: low- and high-performance groups.

#### Time check frequency

To examine whether participants increased clock-checks towards the end of the four-minute period (considered to reflect strategic monitoring) [39, 42], we took the clock-reset-trials for each participant and divided them in one-minute segments. We then counted the number of clock-checks within each one-minute segment and plotted the results using a bar plot with a total of six segments. We used six segments instead of four, as this corresponded to the length of the longest clock-reset-trial. Remember that participants reset the clock at variable times, thus there were no fixed 4-minute clock-reset-trials in the experiment. We performed a repeated measures ANOVA with within factor Time Segments (TS1, TS2, TS3, TS4, TS5, TS6) and between factor Groups (Low-performance, High-performance).

#### Performance in the ongoing task

To explore how the ongoing task was affected by the time-based PM-task, we compared reaction time and accuracy during the PM block relative to performance in the control block. A repeated measures ANOVA included the factors: Task Conditions (Control vs. PM) and Ongoing Trials (Related vs. Unrelated). A between-group factor (Low-performance vs. High-performance) was also included. Note that errors committed during the clock-checks did not contribute to the performance measure, since the appearance of the clock in the screen masked the ongoing task event. Significant interaction effects were examined with one-way ANOVAs and significance level corrected by number of main factor comparisons [43]. Bonferroni correction was used for all post-hoc comparisons. SPSS software was used for behavioural statistical analysis.

### EEG data analysis

#### Single subject level EEG data processing

The EEG data pre-processing and analysis were performed using EEGLAB [44] and custom MATLAB scripts (The Mathworks, Inc.). We first tagged every experimental event with time information using hierarchical event descriptor (HED) tags for analysis of EEG data [45]. Data were visually inspected for bad channel removal. A high-pass finite impulse response (FIR) filter at 1Hz (cut-off frequency, 0.5Hz) and a low-pass FIR filter at 40Hz (cut-off, 45 Hz) were applied to the continuous EEG. The continuous data were then cleaned using the Artifact Subspace Reconstruction method [46, 47], which is an algorithm that removes non-stationary high-variance signals and reconstructs the missing data using a spatial mixing matrix, assuming volume conduction. The algorithm extracts clean sections from the cleanest part of the data and creates a mixing matrix that will be used to interpolate each affected EEG data point. Affected data points were defined based on a variance of 15 or more standard deviations above the variance of uncontaminated EEG data. Infomax independent component analysis (ICA) algorithm [48] was performed to decompose the data into source-resolved activities or independent components (ICs). Equivalent current dipole model estimation of the ICs scalp maps learned by ICA was performed using a Montreal Neurological Institute (MNI) Boundary Element Method (BEM) head model in *DIPFIT*, an EEGLAB plug-in used to fit an equivalent dipole to the scalp projection pattern of each IC [49]. By this means, a total of 288 ICs were obtained from the 24 subjects, after excluding ICs whose dipoles were located outside the brain and those with residual variance of the best-fitting equivalent model dipole of over 15%.

#### Group level data processing: Retrieval mode versus target checking

**Fig 2:**
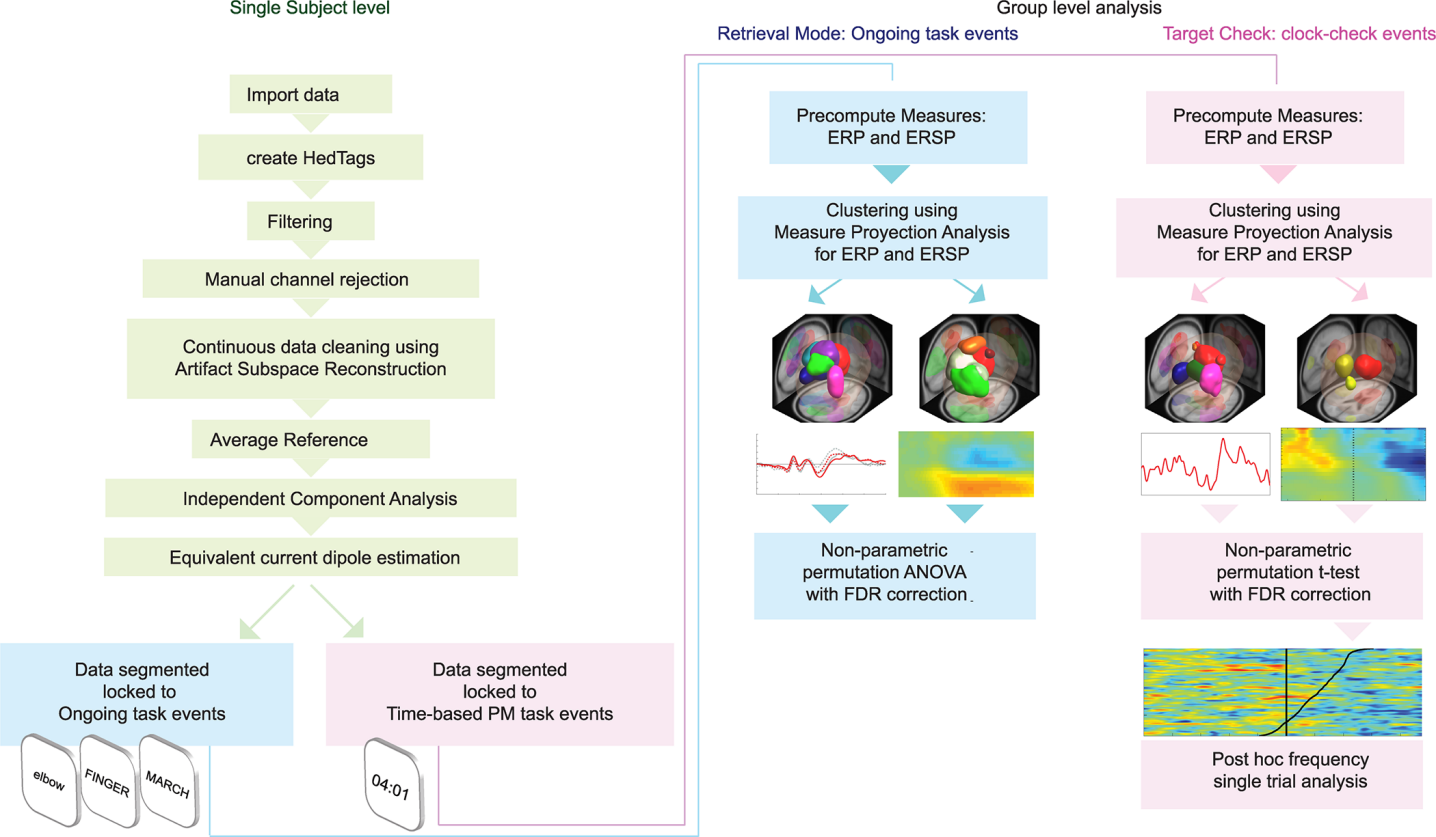
Pipeline for pre-processing and data analysis. Single subject dataset processing was performed on the continuous data (left of the diagram). Two segmented datasets were created from each continuous dataset: blue pipeline aimed at examining brain sources and event related changes locked to the ongoing task stimulus; pink pipeline aimed at examining clock-checks events. The right of the diagram shows group level processing. Measure Projection Analysis was used to find relevant brain areas and ICs statistically similar across all participants during performance of the ongoing task and the time-based PM task. Four different clustering results were obtained. Event-related analysis was performed on the brain activity associated to the brain domain. ERP: Event-Related Potentials, ERSP: Event-Related Spectral Perturbations.

In order to study Retrieval Mode and Target checking separately, we created two new datasets from each pre-processed continuous single subject dataset (Fig 2): for Retrieval Mode, data were segmented locked to ongoing task events into 2-second epochs with one second of baseline. For Target Checking, data were segmented into two-second epochs centred on the clock-checks and clock-resets events. For ERP and ERSP analysis of the time related events we used the whole epoch as baseline. Epochs containing an overlap of ongoing task and time-based PM-task events were not included in the analysis. We used the two types of data segmentation to perform a parallel group analysis (Fig 2): one using ongoing task events only (retrieval mode) and the other using time check events only (target checking).

#### Group level data processing: The problem with ICs group analysis and an alternative solution

One of the issues of averaging channels across participants to perform group analysis is that it equates the location of channels, which may not receive the same combination of source activities in each participant [30], picking up functionally different signals [50] and neglecting the between-subject variance. One way to overcome this difficulty is to use an optimized group-averaging analysis based on source-resolved brain activity (i.e. ICs). However, there is a high variability in location and number of ICs provided by each subject. Measure Projection Analysis (MPA) [51] addresses this issue by using a probabilistic approach.

#### Group level data processing: General description of MPA

As mentioned above, ICs can be modeled as equivalent dipoles [49] located within a standard MNI brain coordinate model. Instead of attributing a single anatomical location to each dipole, MPA finds the probability that one dipole has of being part of different neighboring regions, based on a Gaussian representation of the dipole location. This is what gives this method the character of “probabilistic”. As a result, MPA groups IC dipoles with similar ERPs or ERSPs activities (reflecting similar physiological processes), which have a high probability of being located in the same brain region, henceforth called brain domains. For mathematical details of the algorithms implemented in MPA see [51].

#### Group level data processing: Gaussian representation of IC dipole locations

MPA represents each IC dipole as a 3-D Gaussian distribution within a cubic space grid with 8-mm spacing, situated in a standard MNI brain. MPA only considers dipoles within the MNI brain volume, excluding in the analysis any ICs that cannot be modeled using a single equivalent dipole model. Each Gaussian distribution is located inside the brain model, centered at an estimated dipole location, considering a standard error of 12mm and allowing a maximum of three standard errors. By doing this, it takes into account errors in dipole localization arising through data noise, between-subject variability, error in numerical data decomposition or others [51].

#### Group level data processing: Local convergence value threshold

The next step is to calculate a local convergence value at each voxel location. This consists of an estimate that combines the ERP time-course (or alternatively the ERSP time-frequency image) associated with a given IC and the probability that this IC is truly located at this voxel. Note that MPA only uses one brain measure at a time, e.g. either ERPs or ERSPs in this study, instead of using arbitrarily defined weights to combine the different measures of interest. This reduces the number of parameters assumed in the group analysis, giving a more objective and data-driven way of finding similar brain activities among participants [52], but not excluding human influence at all. MPA assigns significance values to the local convergence values at each brain voxel after bootstrapping, using a surrogate distribution of estimates obtained by randomly re-assigning (with replacement) convergence values to voxel locations. MPA then calculates the local convergence value threshold against the null hypothesis: the convergence value is produced by random brain activity (ERP or ERSP) in the spatial neighborhood. Here, we used a raw statistical consistency threshold of p<0.01. In summary, this step defines a significantly consistent subspace of voxels that is highly unlikely to have occurred by chance.

#### Group level data processing: Creating brain domains

Voxels with significant convergence values are clustered into brain domains using a threshold-based Affinity Propagation clustering method [51]. It starts with a minimum number of clusters (1 or 2) and then, in an iterative process, increases the number of clusters until reaching a maximum “domain exemplar” correlation value. The “domain exemplar” corresponds to a demonstrative sample of the ERP or ERSP activity associated with a potential cluster. In turn, the correlation threshold represents the maximum similarity allowed among “domain exemplars” of different potential clusters. A low correlation (0.2) indicates less similarity between the measure exemplar of the different resulting clusters (low correlation among them). Thus, the iterative process stops when the low correlation value is reached and the subspace of significant convergence values (explained above) is divided into a low number of clusters. In contrast, if the correlation value is high (0.8), the iterative process continues to repeat until reaching the correlation value, resulting in more final clusters within the same subspace. Different correlation thresholds change the granularity of the segmentation of the significant subspace already defined. In sum, a higher correlation threshold produces a greater number of clusters that are more similar to each other. Conversely, lower correlation thresholds produce fewer, more dissimilar and coarser clusters. In this way, the correlation threshold indirectly defines the resulting number of clusters. In the present study we set the correlation threshold to 0.8 (following the criterion used in [51]) and performed subsequent ERP and ERSP activity analysis in only one of the resulting brain domains. We did not inspect scalp level (i.e. electrodes) ERPs or ERSPs.

#### Statistical comparisons

One aim of the statistical analysis was to answer the question of how the ongoing task is affected when it has a time-based prospective memory task embedded. We examined the interference in the processing of ongoing task stimuli during time estimation. Source-resolved ERPs and ERSPs were tested for significant differences using a permutation ANOVA corrected for multiple comparisons using False Discovery Rate (FDR) with alpha level of 0.05. Factors for the ANOVA were: Task Conditions (Control vs. PM) and Ongoing Trials (Related vs. Unrelated). For the ERSP analyses we obtained a matrix of 85 x 20 p-values, corresponding to the time points (0 to 800 milliseconds) and frequencies (3-40Hz), respectively. For the ERP we obtained a vector of 200 p-values, corresponding to the voltage value at each time point (0 to 800 milliseconds). We further examined these differences separately in the two performance groups revealed by the behavioural analysis, using factors: Task Conditions (Control vs. PM) and Groups (Low vs. High-performance). In relation to clock-check events, we performed a permutation t-test with FDR correction (p<0.05) using the factor Groups (Low vs. High-performance).

Note that we used point-by-point statistics (instead of comparing average amplitudes in pre-determined time windows) to elucidate where in the time course of the stimulus processing we find statistical differences. As a consequence, we obtained a large number of values for each ANOVA result. Figures indicate all the time points that remained statistically significant after correction for multiple comparisons, and their corresponding F- or T-value. For non-significant results we report the highest F- or T-value in the text.

For clock-check events, we also performed a *post hoc* trial-to-trial analysis to evaluate whether there was any association between time progression and ERSP changes locked to clock-checks. To build the trial-to-trial visualisation we selected one IC per participant, with the highest probability of being part of the ACC (as we centred the analysis in this brain domain). We obtained the peak frequency within the frequency band 9–15 Hz (as our initial results showed alpha as the frequency of interest for clock-check events) and calculated the power (dB) of the signal on that peak frequency. Then, the power at the peak frequency for each clock check event (trial) was represented by colour-sequence lines and was stacked above each other, resulting in an image with the trial number in the y-axis, time in the x-axis and power in colour code. Events were sorted according to the time they occurred within the clock-reset-trial, indicating changes in the frequency power for each trial in relation to the passage of time; initial trials (or low numbers in the y-axis) occurred early within the clock-reset-trial whereas later trials (high numbers on the y-axis) occurred late within the clock-reset-trial. To statistically assess whether there was a significant change in frequency power, the mean changes in power (dB) were plotted indicating 1% confidence limits according to surrogate data from random windows in the baseline (time courses of the frequency power that fall within the confidence intervals are not significant). No statistical test was performed to evaluate changes across trials (y-axis).

## Behavioural results

### Clock-reset accuracy

**Fig 3:**
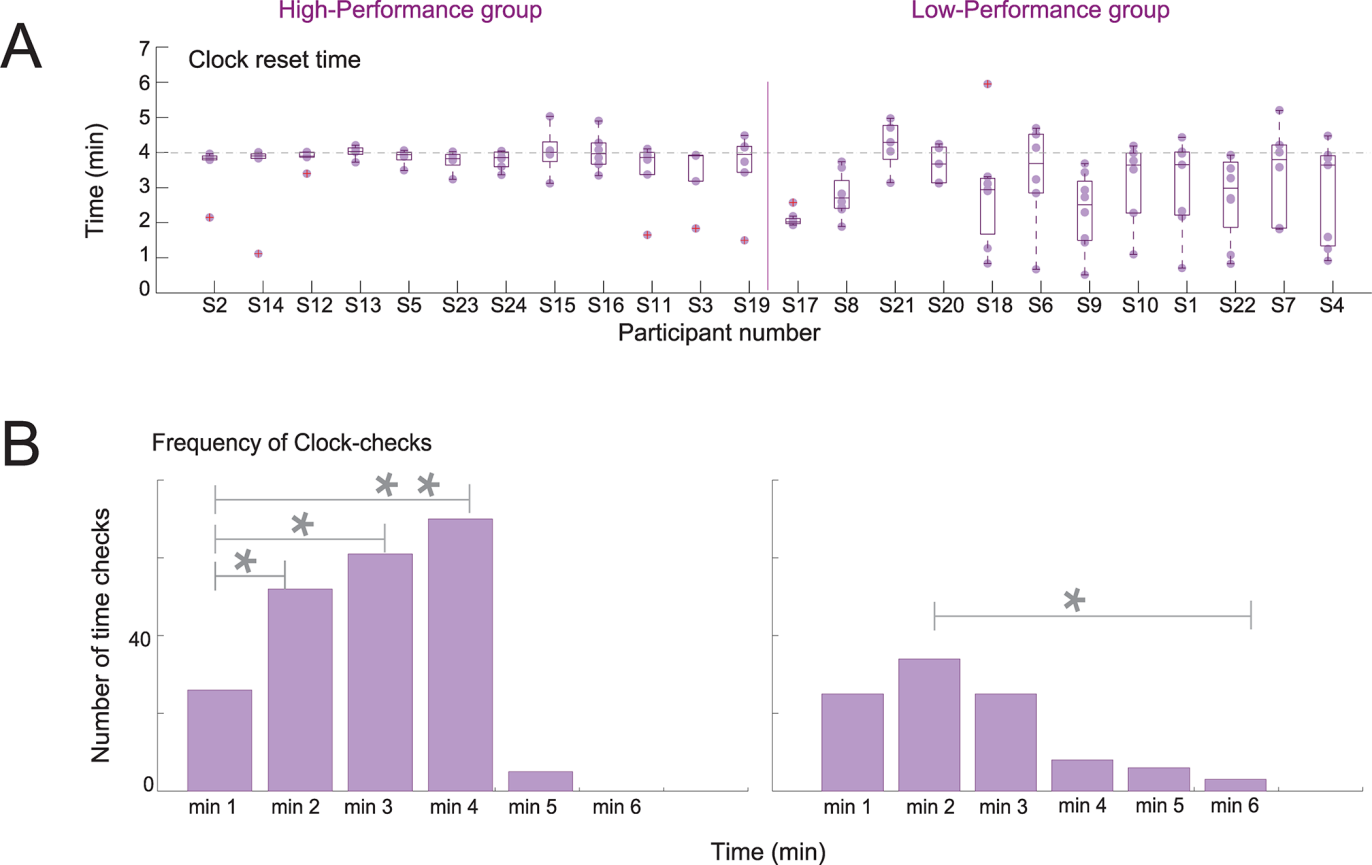
Clock reset time and frequency of time-checks. (A) Clock reset accuracy: Participants were instructed to reset a clock every 4 minutes. Boxplots for each participant in the study show the median and dispersion of clock-reset times. Each circle represents individual clock-resets. The vertical line separates the subjects into two groups. Participants within each group are sorted from smaller to greater interquartile range. (B) Frequency of clock-checks within each clock-reset-trial. High-performers (left) increased number of clock-checks towards the 4-minute target time. Grey lines indicate significant differences, * p < 0.01, ** p < 0.001.

Participants showed a median deviation of 12 seconds from four minutes (target time) (Fig 3A). Participants who reset the clock below the median (with no more than 12 seconds of anticipation or delay) were grouped as ‘high-performers’ and participants who reset the clock above the median (deviation greater than 12 seconds) were considered ‘low-performers’. Table 1 shows general performance for clock-reset accuracy of both groups. To illustrate the variability in the single subject performance we display individual results sorted on two criteria: performance above or below the group median, and according to the Interquartile range (IQR). Participants in the high-performance group had a median close to four minutes with smaller interquartile range (IQR = Q3 –Q1) relative to the low-performance group, meaning that high-performance participants were consistent in resetting the clock close to the four minutes across all clock-reset-trials, see Fig 3A.

**Table 1:** Clock-reset accuracy per group.

	Q3	Median (min)	Q1	IQR
**High Performers (n = 12)**	3.95	3.90	3.85	0.10
**Low Performers (n = 12)**	3.67	3.64	2.82	0.85

Interquartile Range (IQR) = Q3-Q1.

### Time-check frequency

We examined the number of clock-checks within each one-minute segment of the clock-reset-trial using a repeated measures ANOVA, with Time Segment as a within factor and Groups as a between factor. The results showed a significant Time Segment x Group interaction, F(5,110) = 6.932, p < 0.001, indicating that the number of clock-check events during the time segments was different between the groups. The high-performance group checked the clock more frequently as the time progressed, F(5,55) = 22.009, p < 0.001 (Fig 3B). The frequency of clock-checks was significantly higher during ‘time segment 4’ relative to ‘time segment 1’ (p < 0.01). In contrast, the low-performance group showed a homogeneous distribution of clock-checks during the four minutes, the only significant difference being between time segment 2, the highest number of clock-checks, and segment 6, the lowest number of clock-checks, meaning that the low-performance group did not show strategic monitoring of time. The group factor was also significant, indicating that participants from the high-performance group performed more time-checks than the low-performers (M = 2.9, SD = 0.3 versus M = 1.5, SD = 0.3; F(1,22) = 11.205, p < 0.01) (Fig 3C).

### Performance in the ongoing task

To explore how the time-based prospective memory task affected the performance in the ongoing task, accuracy and reaction time were submitted to a two-way repeated measure ANOVA, with the within factor being Task Conditions (Control vs. PM) and the between factor being Groups (High vs. Low-performance), see Table 2. In terms of accuracy, participants decreased their performance in the ongoing task when they also had to undertake the prospective memory task, F(1,22) = 26.224, p < 0.001. There was no interaction effect Task Conditions x Groups, F(1,22) = 0.743, p > 0.05, meaning that the high and low-performance groups decreased accuracy during the ongoing prospective memory task in a similar way.

**Table 2:** Performance in the ongoing task with (PM) and without (control) a prospective memory task embedded.

	Control Task Condition			PM Task Condition		
	Acc	Reaction Time		Acc	Reaction Time	
Group		Related	Unrelated		Related	Unrelated
**High (n = 12)**	94 (3)	677 (75)	785 (117)	92 (3)	685 (87)	714 (78)
**Low (n = 12)**	93 (3)	771 (102)	867 (128)	90 (4)	762 (130)	836 (142)
**Total (n = 24)**	94 (3)	724 (99)	826 (127)	91 (4)	723 (115)	791 (135)

Accuracy (%), Reaction time (ms), standard deviation in parenthesis

To explore reaction times, an additional factor level was added to the ANOVA, Ongoing Trials (Related vs. Unrelated). There was a significant Ongoing Trials x Task Condition interaction, F(1,22) = 11.566 p < 0.01. The post-hoc test showed that reaction times of unrelated items were faster during the PM task condition, t(23) = 1.98, p < 0.05. Faster responses for unrelated events might reflect a practice effect. However, this effect was not further investigated as this difference is marginal and over the corrected significance level 0.025. Reaction times of related items remained the same. There was also a main effect of group; the high-performance group was faster than the low-performance group, F(1,22) = 4.368, p < 0.05. No significant Group x Ongoing Trials x Task Condition interaction was found, F(1,22) = 1.490, p > 0.05, meaning that the high-performance group was faster independently of the experimental conditions.

In summary, the time-based prospective memory task produced a decrease in accuracy of the ongoing task similar for the high- and low-performance groups. Reaction times were not affected, meaning that both groups showed similar behavioural correlates of a retrieval mode. The main behavioural differences between high- and low-performance groups were: the high-performance group showed greater number of time-checks, strategically distributed towards the end of the clock-reset-trial period. This may explain the better results in resetting the clock close to the four-minute target time. In contrast, the low-performance group showed a smaller number of time checks, more evenly distributed across the clock-reset-trial. In addition, the high-performance group showed faster responses in the ongoing task relative to the low-performance group.

## EEG results

### Retrieval mode and time-checking: Brain sources revealed by Measure Projection Analysis

**Fig 4:**
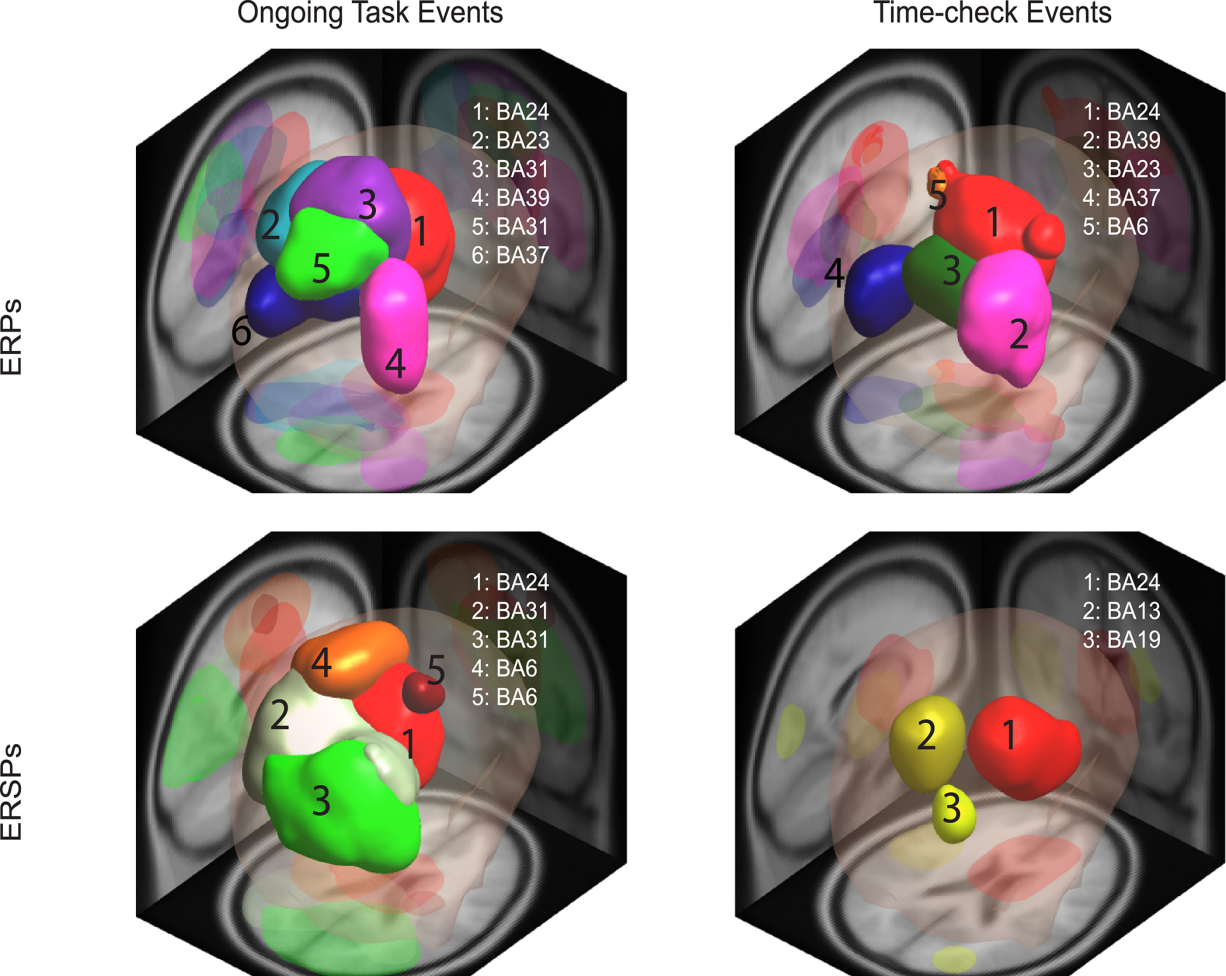
Measure Projection Analysis results. Brain domains revealed by ongoing task events and time-check events (columns), for the two activity measures used in the analysis (rows), ERP and ERSP. Brodmann Areas (BA) are indicated for each result, for details of probability and less probable areas see Table 3. The coloured brain regions represent locations with significant convergence (p<0.01) grouped using a maximum correlation value of 0.8. Note that BA 24 (red domain), here referred to as the MPA-ACC brain domain, is common to all four MPA results.

Pre-processed continuous datasets from each participant were subjected to two different data segmentations in order to find what brain regions and brain activity, revealed by Measure Projection Analysis, were relevant for each monitoring component: retrieval mode and target checking. Measure Projection Analysis uses a probabilistic spatial representation of source localisations, meaning that the results are associated with an estimate of their statistical reliability [51]. Here we present the most probable areas associated with each brain domain for the different Measure Projection Analysis performed. Ongoing task events and clock-check events were analysed separately; for each of them two different analyses were performed to find cortical regions that exhibited consistent ERP and ERSP features across participants. The use of ERP and ERSP separately is important given that different brain areas may be producing the changes observed in each of these measures. The results corresponded to four sets of probable brain regions associated with the performance of the ongoing task and the clock-checks (Fig 4). Details of anatomical areas and Brodmann areas (BA) associated with each brain domain are given in Table 3. Our results showed an anterior brain domain common to the four Measure Projection Analysis results, with the greater probability of being located in BA 24, part of the Anterior Cingulate Cortex. BA 31, part of the Posterior Cingulate Cortex, also appears to be consistent in terms of voltage and frequency changes during the performance of the ongoing task. However, this region was not revealed by clock-check events Measure Projection Analysis results. Our results showed that BA 24 produces the most consistent changes in terms of voltage and frequency associated with the two components of monitoring during the time-based PM task: retrieval mode and time checks. In addition, given the high maximum domain exemplar correlation threshold implemented in the study (0.8), we avoided analysis of neighbouring brain domains due to potential overlap of their corresponding ICs. Therefore, we performed further analysis on ERPs and ERSPs associated with the brain domain with greater probability of being located in the ACC. Hereafter referred to as MPA-ACC brain domain.

**Table 3:** Anatomical areas associated to each domain for ongoing task and time-check events.

	Ongoing Task Events		Time-check Events	
ERP domain	BA	Anatomical Area	BA	Anatomical Area
1	**BA 24 (0.46)**	L Cingulate cortex (0.25)	**BA 24 (0.42)**	L Cingulate Gyrus (0.28)
	BA 31 (0.21)	R Cingulate Gyrus (0.22)	BA 31 (0.22)	R Cingulate Gyrus (0.23)
	BA 23 (0.18)	L Caudate (0.17)	BA 23 (0.20)	L Caudate (0.15)
		L Superior Frontal Gyrus (0.16)		L Superior Frontal Gyrus (0.13)
		R Superior Frontal Gyrus (0.09)		R Caudate (0.09)
				R Superior Frontal Gyrus (0.06)
2	**BA 23 (0.16)**	L Cingulate Gyrus (0.15)	**BA 39 (0.23)**	R Angular Gyrus (0.33)
	BA 31 (0.15)	L Superior Parietal Gyrus (0.14)	BA 13 (0.15)	R Middle Temporal Gyrus (0.15)
	BA 40 (0.15)		BA 41 (0.12)	R Supramarginal Gyrus (0.13)
			BA 22 (0.12)	R Superior Temporal Gyrus (0.10)
3	**BA 31 (0.27)**	L Precentral Gyrus (0.19)	**BA 23 (0.36)**	L Cingulate Gyrus (0.36)
	BA 7 (0.18)	L Cingulate Gyrus (0.14)	BA 30 (0.20)	R Cingulate Gyrus (0.21)
	BA 5 (0.16)		BA 29 (0.19)	
			BA 31 (0.15)	
4	**BA 39 (0.28)**	R Angular Gyrus (0.39)	**BA 37 (0.42)**	L Middle Temporal Gyrus (0.58)
	BA 22 (0.1)	R Middle Occipital Gyrus (0.15)	BA 19 (0.19)	L Superior Temporal Gyrus (0.22)
	BA 31 (0.12)	R Middle Temporal Gyrus (0.14)	BA 41 (0.17)	L Inferior Temporal Gyrus (0.11)
5	**BA 31 (0.57)**	L Precuneus (0.34)	**BA 6 (0.56)**	L Precentral Gyrus (0.85)
	BA 7 (0.37)	R Precuneus (0.32)	BA 4 (0.36)	L Middle Frontal Gyrus (0.15)
6	**BA 37 (0.29)**	L Middle Temporal Gyrus (0.38)		
	BA 19 (0.16)	L Superior Temporal Gyrus (0.12)		
**ERSP domain**				
1	**BA 24 (0.44)**	L Cingulate Gyrus (0.32)	**BA 24 (0.66)**	R Caudate (0.38)
	BA 31 (0.27)	R Cingulate Gyrus (0.24)	BA 33 (0.16)	L Caudate (0.32)
	BA 23 (0.23)	L Caudate (0.14)	BA 25 (0.14)	R Cingulate Gyrus (0.17)
		L Superior Frontal Gyrus (0.13)		L Cingulate Gyrus (0.09)
		R Caudate (0.08)		
		R Superior Frontal Gyrus (0.05)		
2	**BA 31 (0.29)**	L Cingulate Gyrus (0.16)	**BA 13 (0.30)**	L Superior Temporal Gyrus (0.55)
	BA 23 (0.13)	L Superior Parietal Gyrus (0.13)	BA 22 (0.28)	L Middle Temporal Gyrus (0.16)
	BA 30 (0.12)	L Angular Gyrus (0.09)	BA 21 (0.20)	L Insular Cortex (0.15)
		R Cingulate Gyrus (0.07)		L Percentral Gyrus (0.05)
		R Superior Parietal Gyrus (0.06)		
3	**BA 31 (0.25)**	R Superior Parietal Gyrus (0.20)	**BA 19 (0.29)**	R Middle Occipital Gyrus (0.74)
	BA 30 (0.21)	R Lingual Gyrus (0.15)	BA 30 (0.25)	R Superior Occipital Gyrus (0.25)
	BA 18 (0.17)	R Middle Occipital Gyrus (0.10)	BA 18 (0.24)	
			BA 31 (0.21)	
4	**BA 6 (0.51)**	L Precentral Gyrus (0.35)		
	BA 4 (0.17)	L Middle Frontal Gyrus (0.29)		
	BA 3 (0.14)	L Superior Frontal Gyrus (0.23)		
5	**BA 6 (0.66)**	Precentral Gyrus (0.60)		
	BA 4 (0.19)	R Superior Frontal Gyrus (0.29)		
	BA 3 (0.15)	R Middle Frontal Gyrus (0.10)		

Measure Projection Analysis corresponds to a probabilistic approach to cluster brain Independent Component across participants. Here we used ERP and ERSP to identify different Brodmann Areas (BA) relevant for the performance of the task. The table shows the probability (in parenthesis) associated with each BA for ongoing task and time-checks events. The most likely BAs associated with each domain are highlighted in bold. ERP: Event-related potentials. ERSP: Event-related spectral perturbations.

### ERP and ERSP associated to the anterior cingulate cortex brain domain

The following analysis was performed at the brain source level, on the ERP and ERSP activity associated to the MPA-ACC brain domain. In order to explore how the temporal processing of the ongoing task events was affected by holding the time-based delayed intention, we submitted ERP and ERSP measures from the MPA-ACC brain domain to a permutation two-way ANOVA with FDR correction, using the factors Ongoing Trials (Related vs. Unrelated) and Task Conditions (Control vs. PM). For the ERPs the tests were run on 200 data points and, for the ERSPs, on 85x20 data points. Figures show all data points that remained significant after FDR correction at alpha level of 0.05, plus their corresponding F-values. When non-significant effects were found, the highest F-value is indicated in the text.

**Fig 5:**
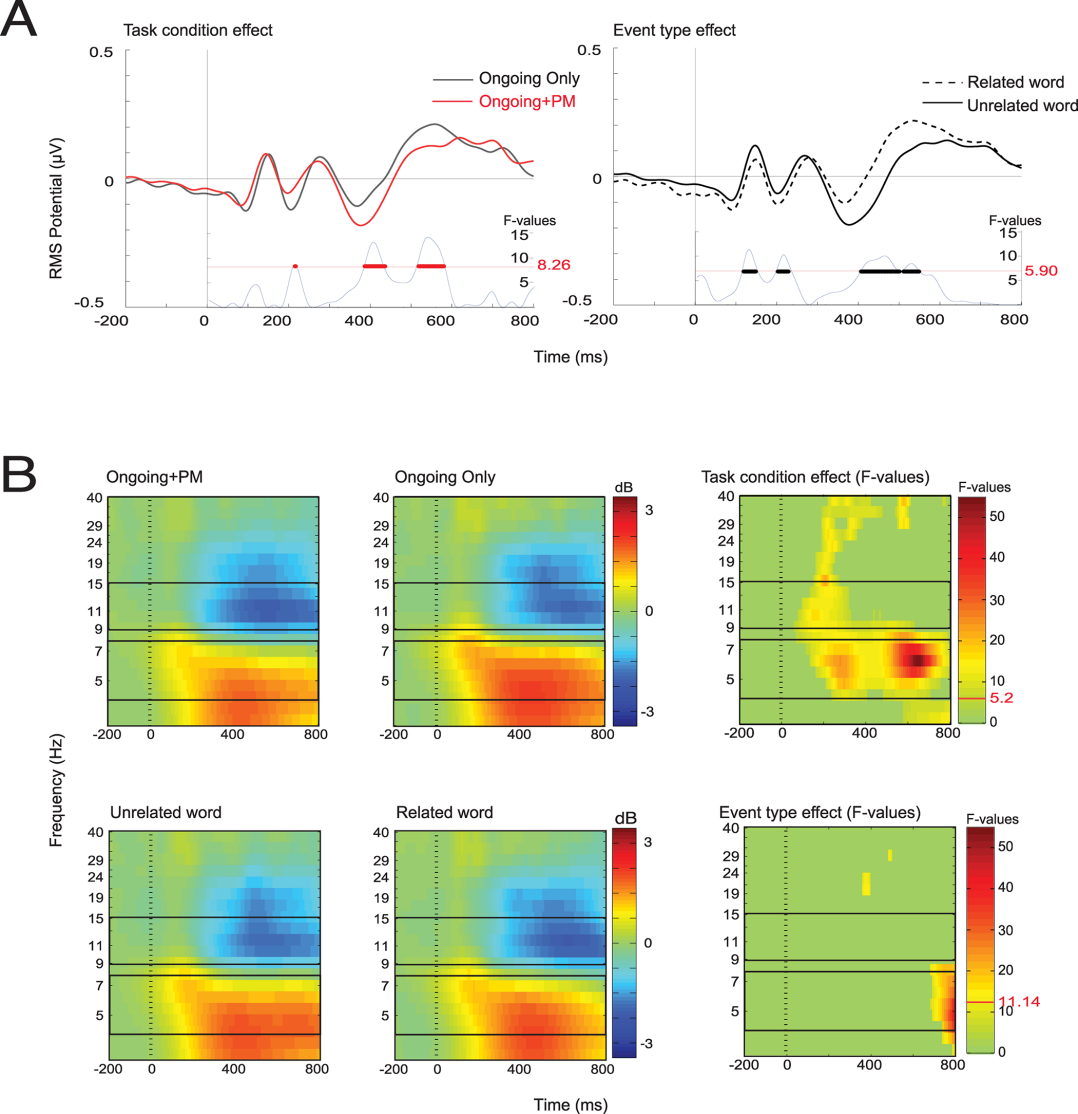
ERP and ERSP locked to ongoing task events, BA 24. (A) The left-hand panel shows the Task Condition effect for ERPs (Event types pooled together). The right-hand panel shows the Ongoing Trials effect (Task Condition pooled together). F-values are shown at the bottom of each panel, the red number indicates the threshold F-value after FDR correction at alpha level of 0.05. (B) Task Condition and Ongoing Trials effects are shown in the top and bottom rows respectively. The box outlines indicate alpha (9–15 Hz) and theta frequencies (5–8 Hz). The most right column shows F-values for the significant difference after FDR correction at alpha level of 0.05. Threshold F-Value for each ANOVA result is indicated in the colour bar to the right of the panel. FDR: False Discovery Rate correction.

We found a significant Ongoing Trials effect: unrelated words showed an enhanced negativity relative to the related words in both control and PM blocks, significant between 350 and 600ms. This N400-like pattern is consistent with the semantic decision participants were required to make in the ongoing task. The comparison between control and PM task conditions (Task Conditions effect) showed significant differences within the same time window (Fig 5A). For the ERSP analysis we found a main Task Condition effect, with stronger alpha (9–15 Hz) suppression and reduced theta power (5–8 Hz) during the PM task condition relative to the Control task condition (Fig 5B). We also observed increased theta power for unrelated words, relative to related words (Ongoing Trials effect), statistically significant around 800 milliseconds. This difference is probably associated with the greater cognitive demand required to correctly identify non-related times. No significant interaction effects were found for either ERPs, F(1,23) = 3.625, FDR corrected p-value > 0.05, or ERSPs, F(1,23) = 8.759, FDR corrected p-value > 0.05, meaning that related and unrelated type events were affected in a similar fashion by the addition of a time-based intention.

**Fig 6:**
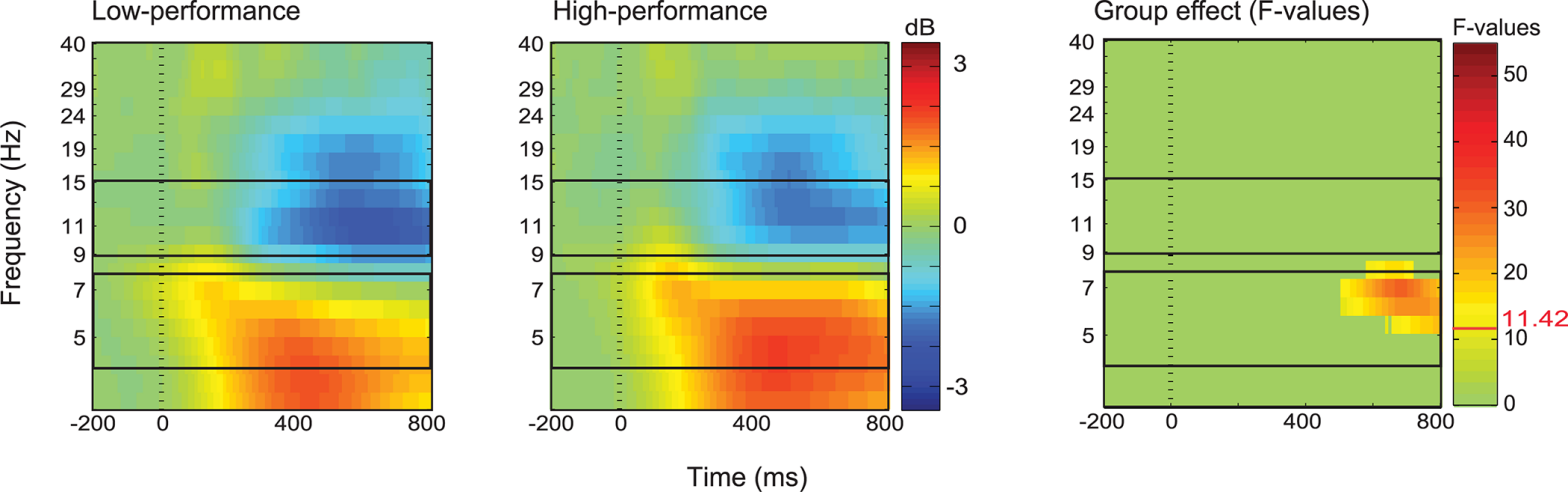
ERSP activity of the anterior cingulate cortex for high- and low-performance groups. Left-hand and middle panels show ERSP to ongoing task events for low- and high-performance groups respectively. Right-hand panel shows a significant group effect (Task conditions pooled together), no significant interaction was found. The box outlines indicate alpha (9–15 Hz) and theta frequencies (5–8 Hz). The most right panel shows F-values for the significant difference after FDR correction at alpha level of 0.05. Threshold F-Value is indicated in the colour bar. FDR: False Discovery Rate correction.

Given that the behavioural analyses showed two different performance groups based on the performance in the time-based PM task (high and low-performers), we further analysed whether the two groups differ on the changes observed in the MPA-ACC brain domain during the PM task condition. We performed a second ANOVA using the factors Group (Low vs. High-performance) and Task Condition (Control vs. PM). Related and unrelated words of the ongoing task were pooled together given that no significant interaction effect was found in the first part of the analysis. We found that low performers showed reduced theta power (5–8 Hz), relative to high performers between 500 and 800 milliseconds (Fig 6). The factor Task Condition remained significant, showing the same differences depicted in Fig 5. However, we found no significant Group x Task Condition interaction, F(1,23) = 8.428, FDR corrected p-value > 0.05, meaning that the difference between groups was independent of whether participants were performing a concurrent time-based task. ERPs showed no significant differences for the factor Group, F(1,23) = 4.283, corrected p-value > 0.05, or the interaction Group x Task Condition, F(1,23) = 5.985, corrected p-value > 0.05. The factor Task Condition showed the same statistically significant differences showed in Fig 5. In sum, both performance groups showed similar neural correlates of a retrieval mode during the performance of the PM task condition.

**Fig 7:**
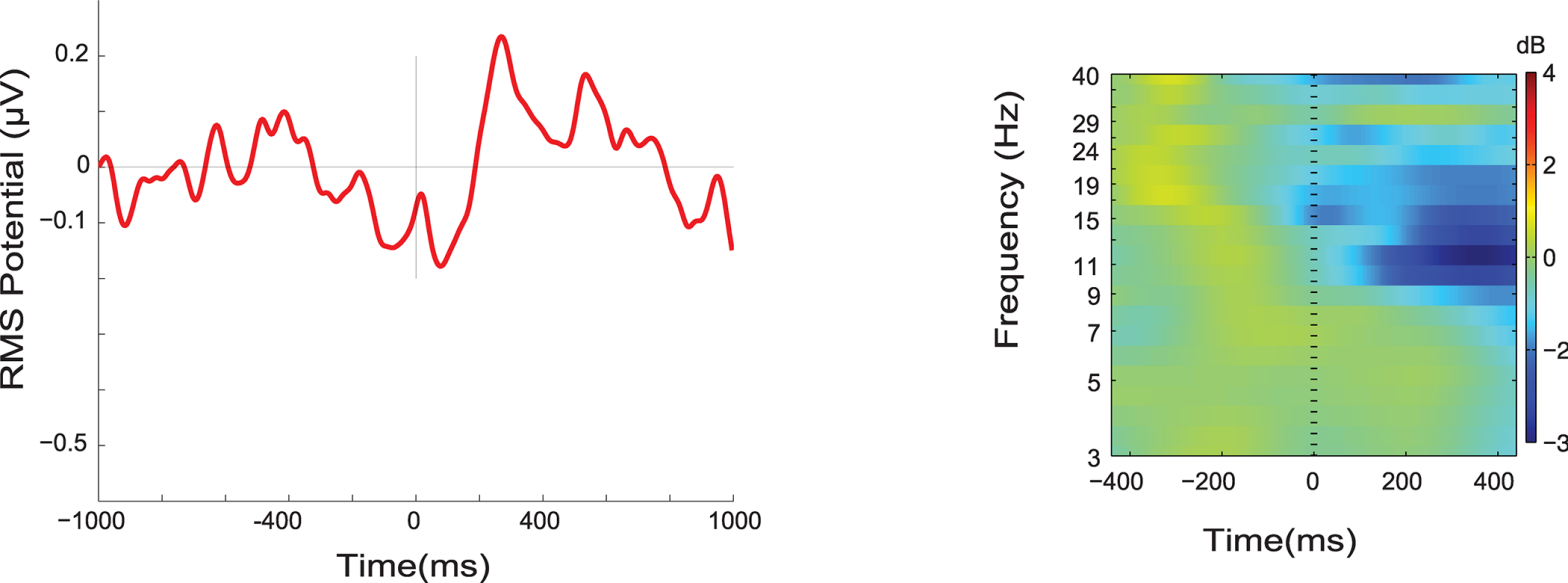
**ERP and ERSP locked to time-checks, BA 24.**(A) ERP shows a negative deflection starting about 400ms before time-check. (B) ERSP shows increased alpha/beta desynchronisation starting right after time-check. Time 0 corresponds to the button press, when participants decided to check the clock.

**Fig 8:**
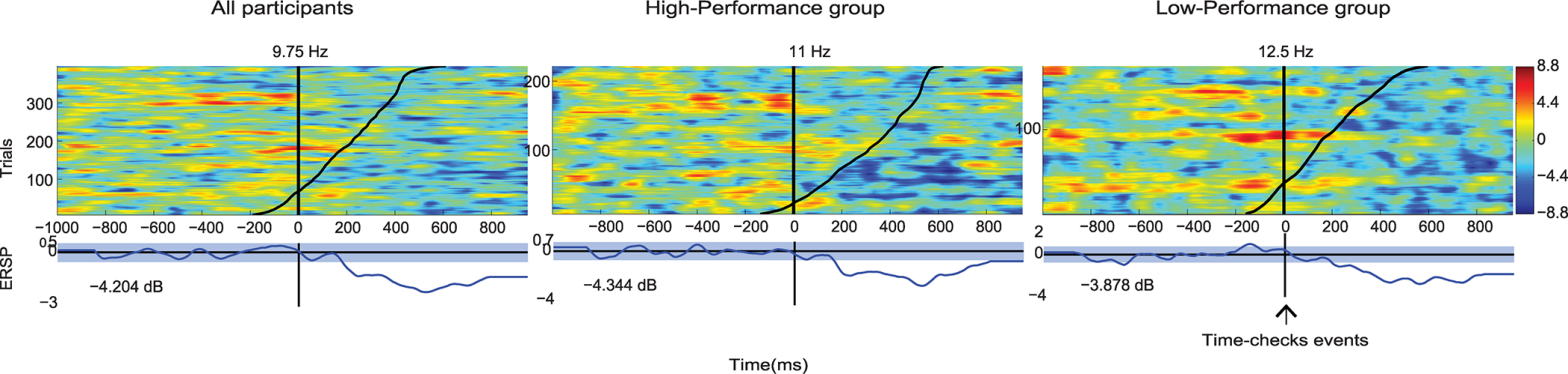
Power trial-to-trial image for clock-check events. Trial-by-trial time courses of alpha power. The power of the signal was calculated based on the peak frequency (indicated at the top of each plot) within the frequency band window 9–15 Hz. Time 0 corresponds to button press to check the clock. Trials are sorted by time along the y-axis (black diagonal line, time is scaled to the figure): low numbers in the y-axis corresponds to trials that occurred early within the clock-reset-trial, whereas trials towards the top of the y-axis occurred closer to the 4-minute target time. The left-hand panel shows all participants. Middle and right-hand panels show high- and low-performance group respectively. Time course of the frequency power is shown at the bottom of each panel, when it falls outside the confidence intervals (blue shading), the decrease in alpha power is statistically significant. Trials are smoothed with a 10-trial moving-average. Note that the number of trials in the y-axis is different for each panel.

ERP and ERSP activity locked to clock-checks is depicted in Fig 7 for the MPA-ACC brain domain. The ERP showed a negative potential starting at about 400ms before a clock-check, which could be an anticipatory negative slow wave associated with preparatory behaviour. The ERSP result shows strong alpha desynchronisation after clock-checks, probably associated with the decision making process after having checked the time. Note that participants were allowed to perform clock-checks when they considered it was most convenient, in order to support the decision of whether or not to reset the clock. We used the factor Group to examine whether there was any difference between high and low performers. No differences were found for either ERPs, F(1,23) = 3.954, corrected p-value > 0.05, or ERSPs, F(1,23) = 3.861, corrected p-value > 0.05. We performed a *post hoc* analysis of the ERSP activity (which seems to be more robust to noise than ERPs) in order to identify whether there was any association between frequency changes and time progression (Fig 8). We observed a clear and statistically significant decrease of alpha power after the clock-check for both groups, but this change was independent of time progression, meaning that similar event related activity was observed when the clock was checked at the beginning of the clock-reset-trial or close to the four-minute target time.

## Discussion

In the present experiment we investigated the neural correlates of retrieval mode and target checking [16] in a time-based prospective memory task. We analysed EEG data in relation to changes in voltage (ERPs) and frequency (ERSP) at a source-resolved level, using ICA and MPA. This study complemented our previous experiments where we investigated neural correlates of strategic monitoring using event-based prospective memory tasks [31].

### Sustained and transient processes contained in the event-related brain activity

A retrieval mode is a sustained mental state or ‘set’ that allows intention retrieval, facilitating the detection of the right moment to perform an intended action [16], whereas target checking is an intermittent process that operates by checking the environment for the occurrence of the prospective memory event [16]. As noted in the introductory section, distinguishing these two components of monitoring is challenging and, in terms of neurophysiological correlates, there is a high variability in the topography, timing and duration of ERP modulations associated with both retrieval mode and target checking. Here we operationalized the retrieval mode as the sustained maintenance of the time-based PM intention during the performance of a non-related ongoing task, and target checking as an intermittent process represented by clock-checks. However, one can question how event related activity, such as ERP or ERSP, could provide a measure of a sustained monitoring process. We assumed that the event-related response is associated with the background ongoing oscillatory activity. For example, previous studies have shown that the power during processing of task stimuli–evaluated using ERSP in the current study–is associated with the absolute frequency power available [53]. In turn, the amplitude of the ERPs reflects stimulus-induced changes associated with phase resetting of ongoing EEG activity [54] and it is not independent from the background oscillatory EEG activity [54, 55]. Thus, even when we are evaluating brain activity locked to ongoing task events, engaging a sustained mental state that allows intention retrieval may produce changes in the oscillatory background EEG activity and in turn this may be reflected in event-related modulations. It is under this logic that we interpret the changes revealed by ongoing task events during the PM block to be neural correlates of a retrieval mode. In this sense, direct association of transient monitoring processes with transient event-related activity (lasting a few milliseconds) and the association of sustained monitoring processes with long lasting event-related modulations (lasting several hundred milliseconds) is not straightforward, something that is reflected in the diverse range of interpretations of neural correlates of retrieval mode [15, 22, 24–26, 29]. West et al. [15] took an interesting approach to studying ERP sustained activity, averaging trials across several seconds including intervals where no stimuli were present. Having said that, future works can explore trial-to-trial changes in order to evaluate consistency of the engagement of a retrieval mode during the ongoing task events performed under the PM task condition, instead of only presenting grand-average ERPs and ERSPs. Additionally, the definition of these two separate components of monitoring may be easier in time-based prospective memory paradigms relative to event-based paradigms. In the present case, the cognitive processes required to perform the time-based intention are different in nature from the ongoing task (monitoring of time progression versus semantic decisions) and the performance of the PM task is independent of the ongoing task, that is, the time-based intention does not require evaluation of the ongoing task events. In contrast, in event-based PM paradigms, the performance of the prospective memory task necessarily requires the evaluation of the ongoing task stimuli to detect the PM task event. Thus, sustained retrieval mode and transient target checking may be mixed in the same event-related brain activity.

### The use of source-resolved brain activity to study prospective memory

We used ICA and Measure Projection Analysis in order to study brain activity from a source level (instead of scalp level activity). The main advantages of this approach were three-fold. First, it allowed the study of EEG brain-imaging with high temporal resolution and improved accuracy for source localisation. Second, we were able to study brain dynamics related to a low number of events because of the increased signal-to-noise ratio [56, 57]. Third, Measure Projection Analysis offers a probabilistic approach by representing IC dipole location as a Gaussian distribution instead of a single point, taking into account errors in dipole localization arising through data noise, between-subject variability, error in numerical data decomposition and others [51]. MPA is a more data-driven approach relative to other clustering methods, because it clusters dipoles using only one type of brain measure at a time (ERP or ERSP) [51], instead of using arbitrarily defined weights to combine the different measures of interest [52]. This reduces the number of parameters assumed in dipole clustering but it does not exclude human influence all together. There are two parameters that are manually set: local convergence value threshold and maximum domain exemplar correlation (see Methods). As with all new methods there are limitations that should be considered (see limitation section).

### Neural correlates of retrieval mode in the time-based PM task

Our results confirmed our hypotheses. We found behavioural and electrophysiological indicators of an active retrieval mode, revealed by: decreased accuracy in the ongoing task during the PM task condition relative to the control condition; greater cognitive effort during the performance of both tasks performed concurrently (greater alpha desynchronization in the MPA-ACC brain domain); and less neural activity associated with the performance of the ongoing task relative to the control condition (decreased theta synchronization in the MPA-ACC brain domain). Note that the strongest differences were observed around 350 and 700 milliseconds, which are probably critical time points associated with word categorisation and response production. However, the lack of counterbalancing of control and PM task conditions deserves consideration; one could argue that decreased accuracy and signs of greater cognitive effort are the result of a fatigue effect, and reduced neural activity associated with the performance of the ongoing task could indicate a practice effect. Considering the behavioural results in the context of the series of experiments we have performed in our laboratory can help allay this concern. In the present and previous experiments [31] we used a fixed order of a control condition before the PM task condition, to avoid contaminating the control block with any long lasting intention maintenance effect evident in other studies [29, 37]. Thus, the same semantic ongoing task was performed concurrently with a time-based PM task (current study), a perceptual event-based PM task (detection of a capitalized letter) and a conceptual event-based PM task (detection of an animal word) [31]. The interference effects were different and specific to the three conditions (Table 4):

- The perceptual event-based PM task did not show behavioural signs of a retrieval mode; accuracy and RTs remained the same during control and PM task conditions.
- In the conceptual event-based PM task, only RTs for unrelated words slowed down during the PM task condition. Note that only unrelated words (and not related words) were also potential PM cues in that context.
- In contrast, the present experiment did not show RT differences between control and PM task condition and only accuracy decreased as a sign of PM interference effect.

**Table 4:** Summary of specific PM interference task effects across three different PM tasks conditions: Perceptual event-based PM task, conceptual event-based PM task and time-based PM task.

	Perceptual	Conceptual	Time-based
**Control**			
RT Related	712 (129)	687 (116)	724 (99)
RT Unrelated	788 (160)	752 (112)*	826 (127)
Accuracy	95 (3)	95 (3)	94 (3)^o^
**Ongoing-PM**			
RT Related	716 (105)	687 (99)	723 (115)
RT Unrelated	801 (137)	791 (108)*	791 (135)
Accuracy	94 (2)	95 (2)	91 (4) ^o^

Accuracy (%) and Reaction Times (ms) per session (standard deviation in parenthesis).

*Significantly slower reaction times for unrelated words of the conceptual PM task condition.

^o^ Significant decrease in accuracy for the time-based PM task condition. No changes in the perceptual PM task condition. For details see [31]. Note that the ongoing task is exactly the same across the three PM task conditions. The only difference is the nature of the PM task.

In terms of the neural correlates of monitoring, our previous study showed an enhanced negativity around 200ms, with common occipital neural generators, for ongoing trials performed during both perceptual and conceptual PM task conditions. We interpreted this as a neural correlate of a retrieval mode. In addition, we found a late correlate of target checking (around 700ms) only for unrelated words in the conceptual PM task. By contrast, in the current study, we found different neural correlates of the retrieval mode. These patterns of results show very specific PM interference effects for each PM task condition performed concurrently with exactly the same semantic ongoing task. If fatigue or practice had produced the effects reported here, one would expect the same behavioural and electrophysiological response in each of the three control-perceptual, control-conceptual and control-time PM tasks. Thus, we conclude that the more plausible interpretation of our results is that the behavioural and electrophysiological changes observed during PM task condition reflect an active retrieval mode that draws resources away from the ongoing task in order to perform the time-based PM tasks.

In terms of the only previous study of the neural correlates of time-based PM tasks [22], our results cannot be directly compared, because that study showed ERP modulations at scalp levels and here we focused on source-resolved event-related activity attributed to the ACC. However, in relation to the idea of an active retrieval mode, Cona et al. [22] observed a sustained positivity over frontal channels as a sign of greater attentional resources devoted to processing ongoing task events. Our approach is conceptually different, as we based our predictions on the idea that the ongoing task events themselves are not relevant for the performance of the time-based PM task, therefore, it would not be necessary to devote more resources to process the ongoing task events. Thus, ERPs would show decreased amplitudes during the PM task condition relative to the control task condition (if fewer resources were available to process ongoing task events). Nevertheless, unlike the ERSP results, our prediction was not clearly supported by our ERP data. Instead of a reduction in amplitude we observed a more negative deflection during performance of the PM task condition relative to the control task condition between 400ms and 600ms, the same time-window when participants were making semantic decisions, in order to correctly respond to the ongoing task (N400-like waveform). Note that to find statistical differences we used point-by-point statistics, meaning that we did not make *a priori* assumptions of differences in specific time windows. One could argue that the differences between both task conditions are related to the attentional modulations exerted through the ACC, but at least in terms of the ERPs it is not clear that this reflects a reduction in cognitive resources.

Our behavioural results also showed a clear distinction between the low and high PM performance groups, in terms of strategic clock-checks. This difference was not reflected in terms of the retrieval mode, given that both groups showed similar decreased accuracy in the ongoing task performed concurrently with the time-based PM task (one could have expected less “monitoring cost” in the high-performance group). Similarly, when we inspected the retrieval mode comparing the PM task conditions with the control condition in the MPA-ACC brain domain, no differences were observed between groups. It is possible that the low number of participants per group (only 12) was underpowered to detect differences, but we are unable to demonstrate this in our study. The only group differences we found were faster response times and greater theta synchronisation for the high-performance group. However, these differences were independent of the task condition, meaning that response times and theta power remained the same when participants performed the ongoing task concurrently with the time-based PM task. The results seem to indicate that even when resources from the MPA-ACC brain domain were devoted to the performance of the time-based PM task, this did not predict a good or bad performance in the time-based task. Thus, the difference between the groups was not associated with the “amount of resources” devoted to the time-based task, but probably with the strategic use of those resources.

### Neural correlates of target checking in the time-based PM task

Our analysis of target checking, represented by clock-checks in this experiment, was exploratory. Areas that have been strongly associated with time estimation (Prefrontral cortex, basal ganglia and cerebellum [58, 59]) were not detected in our study. Instead, our results showed that the anterior brain region that showed activity associated with clock-checks was most probably the ACC. Despite the ACC appearing to be relevant for the performance of clock-checks, we were unable to find a relationship between the passage of time and the MPA-ACC brain domain activity (Fig 8). In terms of the ERP associated with clock-checks we observed a negative deflection around the time check, which may be interpreted as a Contingent Negative Variation (CNV), associated with time-based decision-making process. Different brain regions may be involved in the production of a CNV [60], in particular the anterior cingulate cortex [61]. However, unlike the current study, classical experimental paradigms that produce a CNV use a stimulus (S1) that forewarns the occurrence of a target stimulus (S2). This may be a reason why the negative deflection is not clear in this paradigm, considering that each participant may have implemented different strategies before deciding to check the clock. We suggest that the ACC participates in some type of preparatory/anticipatory process [60, 62] in order to produce a target check response. However, this cannot be clearly deduced from the present study. New experimental paradigms could focus on obtaining a clear negative deflection associated with time checks, given that we have shown that examination of activity attributed to the ACC is feasible using ICA, MPA or other source-resolved clustering methods. On the other hand, the ERSP depicted by clock-checks revealed a strong alpha suppression after the clock-check. Cona et al. [19] suggest in their AtoDI model, that activity in the ACC is associated particularly with the retrieval of intentions. In the current study, it may be that the significant desynchronization observed in the alpha band (Fig 8) after time checking, is associated with retrieval of the time-based intention and post-retrieval decision-making processes; after checking the time participants had to evaluate whether it was time to reset the clock.

### Involvement of the anterior cingulate cortex in the time-based PM task

MPA revealed a brain domain common to both monitoring components (retrieval mode and target checking) and brain measures (ERP time-courses and ERSP time-frequency images): the MPA-ACC brain domain, with greater probability of being located in BA24, part of the Anterior Cingulate Cortex. One of the reasons why we decided to focus only on this region is that due to the high maximum domain exemplar correlation (0.8) set for this study, MPA defines more refined brain domains but neighbouring brain domains can overlap. Thus, to be cautious we performed further analysis only on this region, which showed significant local convergence values for both retrieval mode and target checking. Activation in the anterior cingulate gyrus has been consistently found in different types of time estimation studies [59, 63, 64] and event-based PM tasks [19]. In addition, in our previous study [31] we found that a brain source located in or near the ACC, explained part of the sustained ERP positivity observed over parietal and frontal regions. This sustained positivity was found in two different event-based PM tasks and was related to the retrieval of the intention. This finding supports the AtoDI model [19], which proposes that the ACC has a role in retrieval of the intentions, rather than in their maintenance. Concordantly, the involvement of the ACC in the two types of event-based PM tasks [31] suggests that activity in the ACC may mediate cognitive processes that transcend specific forms of PM tasks. In line with the AtoDI model, the current time-based PM study, showed that the (putative) involvement of the ACC may be more related to attentional control processes implemented during the time related tasks, rather than a time perception function *per se*. But, in contrast to the AtoDI model, we found involvement of the ACC not only during retrieval, but also during the intention maintenance phase. This inconsistency may relate to the fact that the AtoDI model is mostly based on event-based PM experiments, while here we draw our conclusions based on the results of a time-based PM experiment.

Finally, our results are in accordance with the role attributed to the ACC as a regulator of attentional control, signalling the requirement for attentional control exerted by other brain regions [65, 66]. In the current time-based PM tasks, the ACC may have the role of redirecting or distributing attentional resources between the ongoing task and the monitoring of time progression. The neural correlate of a retrieval mode supported by the MPA-ACC brain domain may be reflecting the competing distribution of resources between the ongoing and the time-based PM task. Plus, the activity associated with the time checks may be reflecting post-retrieval decision-making processes mediated by activity in the ACC. In summary, all this suggests that the role of the ACC in time-based PM tasks may be more related to attentional control [65, 66] than to time estimation.

## Limitations

The lack of counterbalanced control and PM blocks is a potential limitation. However, this approach has been used in several studies, with the rationale of having “clean” control blocks [21, 22, 25], avoiding long lasting interference effect of the PM intention into the control block. This design is particularly relevant in the context of studies examining the neural correlates of monitoring. However, some concerns may arise regarding whether the results are produced by fatigue/practice effects rather than the addition of a PM task. One way to overcome this limitation is to use factorial designs that may serve as a control. In this article we have summarised the PM interference effect across three different PM task conditions performed in our laboratory [31]. However, future studies in PM showing robust PM interference effects would benefit from counterbalancing conditions.

We have highlighted some of the advantages of using Measure Projection Analysis. However, as it is a new tool and it represents a probabilistic approach, caution is recommended when interpreting MPA results. For example, as far as we know, there are no empirical studies that show the effect of (or provide practical guidelines for) choosing different local convergence and maximum correlation values, the two variables that are user-specified. In addition, as it is a probabilistic approach, it is actually possible that neighbouring brain domains represent overlapping brain activities. Using a very low maximum domain exemplar correlation may alleviate this, but it would also make brain domains coarser, as the maximum domain exemplar correlation value does not modify the subspace of significant convergence values. In addition, the use of a probabilistic approach makes the issues of localization errors in standard brain models (inherent to any other clustering method) explicit. Thus, caution is always required when attributing brain activity to a brain region using EEG. In the case of MPA, IC dipoles have an associated probability of membership to a brain domain, but there is no certainty of their location. Another issue is that MPA reveals different brain regions depending on the particular measure used (ERP or ERSP). This does not suggest a contradiction, given that voltage and frequency changes may be sensitive to different phenomena in the brain. But it can make results difficult to interpret. Further empirical and theoretical development of MPA is required in order to provide a more extensive interpretation of our results. Although the methods we have implemented here allow improved spatial resolution, it is important to keep in mind that they are not characterized by high spatial resolution. Better head models should be used for dipole fitting, as there are known margins of error in the localisation of the independent components [67].

In time-based PM paradigms using source localization methods, one might expect to find activity in frontal regions of the brain, such as BA 10, which has been shown to be key for performance of prospective memory tasks or other prefrontal regions associated with time estimation. However, the brain source analysis did not consistently show any frontal area other than the MPA-ACC brain domain. It may be possible that our experimental paradigm in conjunction with the methods implemented were not sensitive enough to study activity in frontal regions. By no means do we exclude the relevance and the involvement of those areas.

Finally, in terms of the experimental paradigm, the requirement for time estimation can vary among different prospective memory tasks, depending on the availability of external indicators of time (for example, to have a clock continuously visible). It may be that the always-available option of checking the clock could have reduced the demand on internal time estimation. To further explore the involvement of time estimation, as a component of monitoring in time-based prospective memory task, would require using tasks that allow different degrees of internal time estimation.

## Conclusion

The ACC is an important brain region to explore in relation to brain dynamics associated with the performance of time-based prospective memory tasks using EEG. Behavioural and neural (ERPs and ERSPs) changes observed during the Ongoing plus PM task condition, with their source in or close to BA 24, support the idea that the ACC exerts attentional modulation during the maintenance of a time-based intention. Thus, during the retrieval mode the involvement of the ACC may be related to strategic distribution of attentional resources between the ongoing task and the time-based decision. The two performance groups, identified based on the performance in the time-based PM task, showed similar behavioural and neural correlates of a retrieval mode, meaning that the retrieval mode is active across the participants independently of their level of performance in the time-based PM task. While the brain domain likely located in the ACC was consistently involved during clock-checks, its role does not seem to be associated with time perception processing but it is probably involved in anticipatory and decision making processes. Finally, the likely involvement of the ACC in both aspects of time-based PM monitoring may reflect different functions that have been attributed to it and reflect the ubiquitous involvement of the ACC in highly cognitive demanding tasks.
